# Dementia Risk Factors Modify Hubs but Leave Other Connectivity Measures Unchanged in Asymptomatic Individuals: A Graph Theoretical Analysis

**DOI:** 10.1089/brain.2020.0935

**Published:** 2022-02-11

**Authors:** Hannah Clarke, Eirini Messaritaki, Stavros I. Dimitriadis, Claudia Metzler-Baddeley

**Affiliations:** ^1^ Cardiff University Brain Research Imaging Centre (CUBRIC), School of Psychology, Cardiff University, Cardiff, United Kingdom.; ^2^ School of Medicine, UK Dementia Research Institute, Cardiff University, Cardiff, United Kingdom.; ^3^ BRAIN Biomedical Research Unit, School of Medicine, Cardiff University, Cardiff, United Kingdom.; ^4^ Neuroinformatics Group, Cardiff University Brain Research Imaging Centre, School of Psychology, Cardiff University, Cardiff, United Kingdom.; ^5^ Division of Psychological Medicine and Clinical Neurosciences, School of Medicine, Cardiff University, Cardiff, United Kingdom.; ^6^ School of Psychology, Cardiff University, Cardiff, United Kingdom.; ^7^ Neuroscience and Mental Health Research Institute, School of Medicine, Cardiff University, Cardiff, United Kingdom.; ^8^ MRC Centre for Neuropsychiatric Genetics and Genomics, School of Medicine, Cardiff University, Cardiff, United Kingdom.

**Keywords:** Alzheimer's disease, graph theoretical analysis, hubs, risk factors

## Abstract

***Background:*** Alzheimer's disease (AD) is the most common form of dementia with genetic and environmental risk contributing to its development. Graph theoretical analyses of brain networks constructed from structural and functional magnetic resonance imaging (MRI) measurements have identified connectivity changes in AD and individuals with mild cognitive impairment. However, brain connectivity in asymptomatic individuals at risk of AD remains poorly understood.

***Methods:*** We analyzed diffusion-weighted MRI data from 161 asymptomatic individuals (38–71 years) from the Cardiff Ageing and Risk of Dementia Study (CARDS). We calculated white matter tracts and constructed whole-brain, default mode network (DMN) and visual structural brain networks that incorporate multiple structural metrics as edge weights. We then calculated the relationship of three AD risk factors, namely Apolipoprotein-E ɛ4 (*APOE4*) genotype, family history of dementia (FH), and central obesity (Waist-Hip-Ratio [WHR]), on graph theoretical measures and hubs.

***Results:*** We observed no risk-related differences in clustering coefficients, characteristic path lengths, eccentricity, diameter, and radius across the whole-brain, DMN or visual system. However, a hub in the right paracentral lobule was present in all the high-risk groups (FH, *APOE4*, obese), but absent in low-risk groups (no FH, *APOE4*-ve, healthy WHR).

***Discussion:*** We identified no risk-related effects on graph theoretical metrics in the structural brain networks of cognitively healthy individuals. However, high risk was associated with a hub in the right paracentral lobule, a medial fronto-parietal cortical area with motor and sensory functions. This finding is consistent with accumulating evidence for right parietal cortex contributions in AD. If this phenotype is shown to predict symptom development in longitudinal studies, it could be used as an early biomarker of AD.

**Impact statement:**

Alzheimer's disease (AD) is a common form of dementia that to date has no cure. Identifying early biomarkers will aid the discovery and development of treatments that may slow AD progression in the future. In this article, we report that asymptomatic individuals at heightened risk of dementia due to their family history, Apolipoprotein-E ɛ4 genotype, and central adiposity have a hub in the right paracentral lobule, which is absent in low-risk groups. If this phenotype were to predict the development of symptoms in a longitudinal study of the same cohort, it could provide an early biomarker of disease progression.

## Introduction

Alzheimer's disease (AD) is one of the major causes of dementia that affect 10% of individuals older than the age of 65. In the United States, over 1 million individuals per year will be affected by AD by 2050 (Hebert et al., 2013). A recent review by the Lancet Commission concluded that almost half of the dementia cases might be prevented or delayed by modifying 12 risk factors (Livingston et al., 2020). It emphasized the importance of improving the early detection of individuals at risk of developing AD so that preventative therapeutics can be discovered and developed in the future. It is therefore important to gain a better understanding of how AD risk factors affect the structure of the brain in healthy individuals and how risk-related effects differ from those of healthy aging.

The human brain has been characterized as a network of cortical and subcortical areas (network nodes) that communicate with each other via white matter tracts (connections or edges) that carry neuronal signals (Bullmore and Bassett, 2011; Rubinov and Sporns, 2010). Structural networks can be derived from diffusion-weighted magnetic resonance imaging (dMRI) data via tractography (Basser et al., 2000; Mukherjee et al., 2008a,b), and are represented mathematically by graphs. Graph theory can then be used to quantify the local and global organizational properties of the brain's structural connectome (Bullmore and Sporns, 2009).

Graph theoretical analyses of brain networks have provided insight into the effect of AD on the brain's connectivity (Dai et al., 2019; John et al., 2017; Lo et al., 2010). More specifically, there is strong evidence that even though AD pathology is initially present in localized brain areas, it still affects the whole brain as a network. It is, therefore, possible that people at risk of developing AD could show alterations in their structural brain networks and their graph theoretical metrics before developing the disease. This implies that investigations into possible relationships between AD risk factors and graph theoretical metrics of structural brain networks could provide biomarkers that signal disease onset or track disease progression.

In the present study, we used graph theory to characterize the mesoscale of structural brain networks for the whole-brain connectome and for a system that is known to be affected in AD, namely the default mode network (DMN), as well as the visual network as a control (Badhwar et al., 2017), in 161 cognitively healthy individuals from the Cardiff Ageing and Risk of Dementia Study (CARDS) (38–71 years) (Coad et al., 2020; Metzler-Baddeley et al., 2019a,b; Mole et al., 2020a,b) with different risk factors for AD. The risk factors investigated were Apolipoprotein-E ɛ4 (*APOE4*), family history of dementia (FH), and central obesity as assessed with the Waist-Hip-Ratio (WHR). A statistical framework was followed to reveal potential differences in the structural network organization between groups of aggregated risk levels. Our hypothesis was that individuals at the highest risk of dementia, that is, obese *APOE4* carriers with a FH, compared with those at lowest risk, that is, normal-weighted noncarriers without a family history, would have altered integration and segregation parameters (increased characteristic path lengths, decreased clustering, etc.). In our exploratory analysis of hubs, we aimed to identify any highly interconnected nodes that consistently differed between low- and high-risk group (FH vs. no FH, *APOE4* carrier vs. noncarrier, obese vs. healthy WHR).

## Materials and Methods

Details of the CARDS procedures have been previously published (Coad et al., 2020; Metzler-Baddeley et al., 2019a,b; Mole et al., 2020a,b) and hence are only briefly described in the following. The CARDS was approved by the School of Psychology Research Ethics Committee at Cardiff University (EC.14.09.09.3843R2) and all participants provided written informed consent.

### Participants

Individuals between the ages of 38 and 71 were recruited from the local community via Cardiff University community panels, notice boards, and poster advertisements. Exclusion criteria included a history of neurological and/or psychiatric disease, severe head injury, drug or alcohol dependency, high-risk cardioembolic source, or known significant large-vessel disease. MRI screening criteria were fulfilled by 166 participants. Table 1 summarizes their demographic background, and information about their genetic and lifestyle risk variables. Depression was screened for with the Patient Health Questionnaire (PHQ-9) (Kroenke et al., 2001), verbal intellectual function was assessed with the National Adult Reading Test (NART) (Nelson, 1991), and cognitive impairment with the Mini Mental State Examination (MMSE) (Folstein et al., 1975). One participant was excluded after assessment of the MMSE score (MMSE = 26). Four participants had missing data, and thus, the final analysis had a sample size of 161.

**Table 1.** Participant Demographics

	Mean (σ)			
Age	55.76 (8.22), range: 38–71			
Males	71/165			
Years of education	16.55 (3.32), range: 9.5–26			
FH	59/163			
*APOE4* carriers	64/164			
WHR obese	102/165			

	N (M)	N (F)	Mean age, M (σ)	Mean age, F (σ)
Demographics broken down by risk factor				
No FH, No *APOE4*, Healthy weight	4	16	53.75 (4.03)	53.69 (8.68)
FH, No *APOE4*, Healthy weight	0	13	—	53.85 (6.87)
No FH, *APOE4*, Healthy weight	3	16	49.00 (7.21)	52.88 (9.64)
No FH, No *APOE4*, Obese	18	21	56.17 (8.74)	56.05 (7.68)
FH, *APOE4*, Healthy weight	4	6	59.00 (2.45)	59.83 (5.19)
No FH, *APOE4*, Obese	20	6	54.00 (9.61)	58.67 (6.56)
FH, No *APOE4*, Obese	17	10	58.71 (7.71)	56.60 (9.35)
FH, *APOE4*, Obese	5	3	57.00 (8.22)	62.00 (7.94)

This table lists the demographics (age, years of education and sex) of the participants who took part in this study, and splits M and F data by risk factor group. Mean age and years of education, accurate to 2 decimal places, are quoted with standard deviations reported in brackets (σ).

*APOE4*, Apolipoprotein-E ɛ4; F, female; FH, family history of dementia; M, male; WHR, waist/hip ratio.

### Assessment of risk factors

Participants gave saliva samples with the Genotek Oragene-DNA kit (OG-500) for *APOE* genotyping. *APOE* genotypes ɛ2, ɛ3, and ɛ4 were determined by TaqMan genotyping of single-nucleotide polymorphism (SNP) rs7412 and KASP genotyping of SNP rs429358 (Metzler-Baddeley et al., 2019a). Genotyping was successful for 164 of the 165 participants. In addition, 163 participants provided information about their FH, that is, whether a first-grade relative was affected by AD, vascular dementia, or any other type of dementia. We also obtained the number of years spent in education for the 164 participants, to include as a covariate in this analysis (Table 1).

Participants' waist and hip circumferences were measured to calculate the waist/hip ratio (WHR). Central obesity was defined as a WHR ≥0.9 for men and ≥0.85 for women (Table 1). Other metabolic risk factors were self-reported in a medical history questionnaire [see for details Mole and collegues (2020a) *Neurobiology of Aging*] but were not included in the present analysis.

### MRI data acquisition

MRI data were collected on a 3T MAGNETOM Prisma clinical scanner (Siemens Healthcare, Erlangen, Germany) (Coad et al., 2020; Metzler-Baddeley et al., 2019a,b; Mole et al., 2020a) at the Cardiff University Brain Research Imaging Centre (CUBRIC). A 3D magnetization-prepared rapid gradient-echo sequence was used to acquire T_1_-weighted anatomical images with the following parameters: 256 × 256 acquisition matrix, TR = 2300 ms, TE = 3.06 ms, TI = 850 ms, flip angle θ = 9°, 176 slices, 1 mm slice thickness, 1 × 1 × 1 mm isotropic resolution, FOV = 256 mm, and acquisition time of ∼6 min.

Diffusion-weighted magnetic resonance images were acquired with high angular resolution diffusion imaging (HARDI) (Tuch et al., 2002) using a spin-echo echo-planar dual-shell HARDI sequence with diffusion encoded along 90 isotropically distributed orientations (Jones et al., 1999) (30 directions at *b* = 1200 sec/mm^2^, 60 directions at *b* = 2400 sec/mm^2^) as well as 6 nondiffusion-weighted images with dynamic field correction using the following parameters: TR = 9400 ms, TE = 67 ms, 80 slices, 2 mm slice thickness, 2 × 2 × 2 mm voxel, FOV = 256 × 256 × 160 mm, GRAPPA acceleration factor = 2, and acquisition time of ∼15 min.

### HARDI data processing and whole-brain tractography

Diffusion-weighted imaging data processing has been previously detailed in Coad and colleagues (2020), Metzler-Baddeley and colleagues (2019a,b), and Mole and colleagues (2020a,b). In brief, dual-shell data were split and *b* = 1200 and 2400 sec/mm^2^ data were corrected separately for distortions induced by the diffusion-weighted gradients and motion artifacts in ExploreDTI (v4.8.3) (Leemans et al., 2009). Echo planar imaging-induced geometrical distortions were corrected by registering the diffusion-weighted image volumes to the T_1_-weighted images (Irfanoglu et al., 2012).

Outliers in the diffusion data were identified with the RESDORE algorithm (Parker, 2014). Whole-brain tractography was performed with the damped Richardson/Lucy algorithm (dRL) (Dell'Acqua et al., 2010) on the 60 direction, *b* = 2400 sec/mm^2^ HARDI data for each data set in single-subject space using in-house software (Parker, 2014) coded in MATLAB (The MathWorks, Natick, MA). Fiber tracts were reconstructed by estimating the dRL fiber orientation density functions (fODFs) at the center of each image voxel with seed points positioned at the vertices of a 2 × 2 × 2 mm grid superimposed over the image. At each seed point, the tracking algorithm interpolated local fODF estimates and then propagated 0.5 mm along orientations of each fODF lobe above a threshold of a peak amplitude of 0.05. Individual streamlines were then propagated by interpolating the fODF at their new location and by propagating 0.5 mm along the minimally subtending fODF peak. This process was repeated until the minimally subtending peak magnitude fell below 0.05 or the change of direction exceeded an angle of 45°. Tracking was subsequently repeated in the opposite direction from the initial seed point. Streamlines with lengths outside a range of 10 to 500 mm were removed.

### Generating integrated weighted structural brain networks: whole-brain analysis

Whole-brain tractography maps were used in ExploreDTI v4.8.6 (Leemans et al., 2009) to create connectivity matrices that describe the structural connectome mathematically. Network nodes were defined according to the automated anatomical labeling (AAL) atlas (Tzourio-Mazoyer et al., 2002) using the 90 cortical and subcortical areas of the cerebrum. The edges of the networks were the tractography-reconstructed tracts: all edges between brain areas not connected by tracts were therefore equal to zero. This process resulted in sixteen 90 × 90 connectivity matrices, the edges of each quantifying if there was a tract or not, number of streamlines between two nodes, percentage of tracts (PS) between two nodes, average tract length (ATL), Euclidean distance (ED), density of tracts, tract volume (TV), mean diffusivity (MD), axial diffusivity (AxD), radial diffusivity (RD), fractional anisotropy (FA), second and third eigenvalue of the diffusion tensor, linear anisotropy, planar anisotropy, and spherical anisotropy.

The above mentioned metrics were chosen because they could reflect the signal transport and integration abilities of the structural connectome (Messaritaki et al., 2021). In addition, the strength of the structural connectivity between brain areas depends on the metric used to weight the network edges. As a result, the network measures derived via the graph theoretical analysis depend on the connectivity matrix used—that is, which of the above metrics we chose as an edge weight. We have recently shown that this ambiguity can be solved by linearly combining nine normalized metrics (number of tracts, PS, ATL, ED, density, TV, MD, RD, and FA) into a single graph (Dimitriadis et al., 2017b) and thresholding the subsequent graphs using an orthogonal minimal spanning tree scheme (Dimitriadis et al., 2017a). This protocol creates connectivity matrices that combine the information from the included metrics in a data-driven manner, so that the maximum information from all metrics is retained in the final graph; these are termed integrated graphs. The thresholding step can be applied in dense matrices, resulting in a topographically filtered integrated weighted structural brain network. The network and nodal reliability of such integrated graphs was improved beyond that of the nine individual metrics (Dimitriadis et al., 2017b). In addition, they were shown to have very good discrimination capability in a binary classification problem (Dimitriadis et al., 2017b), and to exhibit good scan/rescan reliability (Messaritaki et al., 2019a,b). A recent study demonstrated that community partitions and provincial hubs are highly reproducible in a test/retest study when structural brain networks were constructed with the integrated approach (Dimitriadis et al., 2020). For those reasons, we created integrated weighted brain networks instead of pursuing a single-metric structural connectivity matrix.

**FIG. 1.**
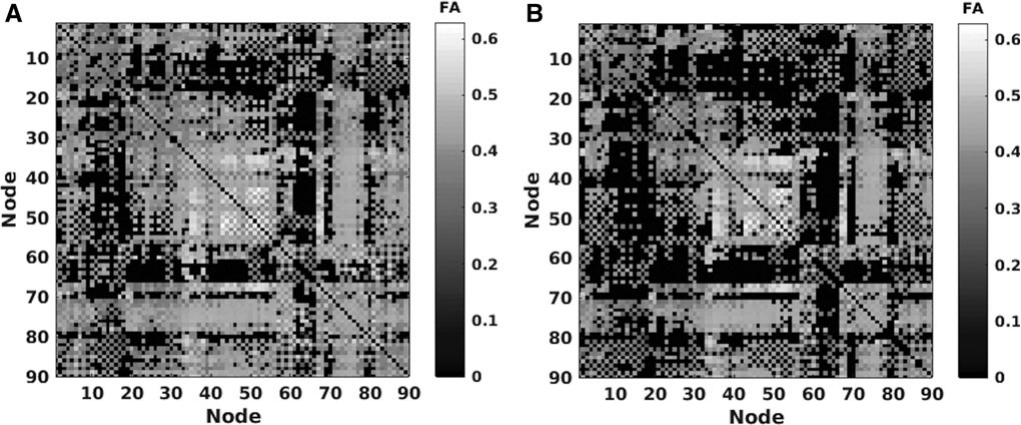
An example of the conservative threshold added to all dMRI connectivity matrices. **(A)** FA connectivity matrix for one participant before thresholding. **(B)** After a conservative threshold of five streamlines was applied to FA for the same participant. dMRI, diffusion-weighted magnetic resonance imaging; FA, fractional anisotropy.

To reduce the number of false positives possibly resulting from the tractography, we set to zero all edges in the structural connectivity matrices that corresponded to tracts with fewer than five streamlines (excluding ED as this is a biological metric and has a value regardless of the number of streamlines). All subsequent analyses were performed on these thresholded connectivity matrices (Fig. 1).

To decide which metrics to combine into the integrated weighted structural brain network, we calculated the intercorrelation coefficients (Corrcoef, MATLAB R2015a) between the number of streamlines (NS), PS, ATL, ED, density of streamlines (SLD), TV, MD, RD, AxD, and FA, see Table 2. In addition, we performed a multicollinearity test (Collintest, MATLAB R2015a) in an endeavor to eliminate metrics representing redundant information within our integrated graphs. After excluding highly correlated and multicollinear metrics, the remaining metrics were integrated into a single graph via a linear graph-distance combination (Dimitriadis et al., 2017b).^1^

**Table 2.** Abbreviations Used for the Diffusion-Weighted Magnetic Resonance Imaging Metrics

Name of dMRI metric	Abbreviation
Number of streamlines	NS
Percentage of tracts	PS
Average tract length	ATL
Euclidean distance	ED
Streamline density	SLD
Tract volume	TV
Mean diffusivity	MD
Radial diffusivity	RD
Axial diffusivity	AxD
Fractional anisotropy	FA

This table defines the abbreviations used throughout the article for each of the dMRI metrics.

dMRI, diffusion-weighted magnetic resonance imaging.

### Calculating network measures from integrated graphs

The resulting graphs were weighted and undirected. Using the MATLAB Brain Connectivity Toolbox (Rubinov and Sporns, 2010), we calculated the following metrics:

- Clustering coefficient: A measure of how interconnected nodes are (averaged across all nodes)
- Characteristic path length: The average minimum number of connections to link two nodes
- Eccentricity: Maximum shortest distance between one node and all others (averaged across all nodes)
- Radius: Minimum eccentricity
- Diameter: Maximum eccentricity
- Global efficiency: Inverse of the characteristic path length^2^

Network measures were examined for multicollinearity using Belsley collinearity diagnostics (Collintest, MATLAB R2015a) to ensure that only unique predictors were included in our analysis. The remaining network measures were analyzed using the multivariate general linear models described below. We were also interested in identifying potential interactions between our risk factors.

### Subnetwork analysis

**FIG. 2.**
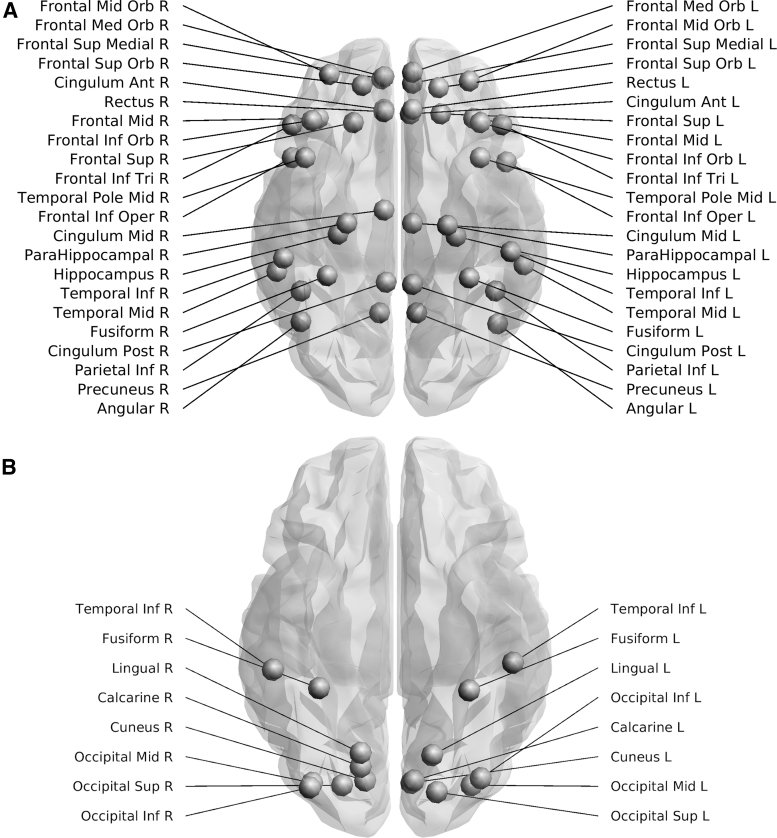
Nodes included in the subnetwork analysis for the DMN and visual system. The top figure **(A)** shows the 44 nodes included in the DMN adapted from Power et al. (2011), whereas the bottom figure **(B)** shows the 16 nodes included in the visual network adapted from Power et al. (2011). Images were created using ExploreDTI v4.8.6. DMN, default mode network.

As AD preferentially impacts the DMN, we repeated the analysis for this subnetwork by adapting the AAL atlas (Tzourio-Mazoyer et al., 2002) based on the data from Power and colleagues (2011). The DMN graphs comprised 22 nodes from each hemisphere encompassing the frontal, temporal, and parietal lobes, including the precuneus, cingulate gyrus, and hippocampus (Fig. 2). To investigate if any changes were specific to the DMN, we analyzed a separate control subnetwork—the visual system (Wang et al., 2012), by adjusting the regions of interest specified in Power and colleagues (2011). The resulting integrated weighted structural brain networks were composed of 16 nodes from the left and right hemispheres: inferior temporal gyrus, fusiform gyrus, superior/middle/inferior occipital gyrus, lingual gyrus, cuneus, calcarine fissure, and the surrounding cortex (Fig. 2).

### Hub analysis

Hubs are nodes of a network that are highly connected to other nodes and act as bridges that facilitate the transfer of signals in the brain, contributing to its integration abilities (van den Heuvel and Sporns, 2013). Crucially, hubs appear to play a role in AD (Buckner et al., 2009). We split the cohort into risk factor groups—positive (*N* = 59) versus negative family history (*N* = 104), *APOE4* carriers (*N* = 64) versus noncarriers (*N* = 100), centrally obese (*N* = 102) versus healthy weight (*N* = 63)—to explore whether hubs changed as a function of risk factor profile in healthy individuals. Hubs were identified across the whole brain for each participant by ranking nodal betweenness centrality and strength, where higher scores indicate hubs. In addition, nodal local efficiency and clustering coefficients were ranked, with smaller values indicating hubs. A node was defined as a hub when it was in the top 20% for global measures and the lowest 20% for local measures. Using replicator dynamics (Dimitriadis et al., 2010; Neumann et al., 2005), hubs that were consistently present across the individual risk factor cohorts were determined.^3^ This analysis was then repeated using data from the DMN and visual subnetworks to identify internally important nodes.

### Statistical analyses

In SPSS v26 (IBM Corp., 2019), we performed multivariate general linear models with factors of *APOE4* carrier/noncarrier, FH/no family history, and WHR obese/healthy on dependent variables; mean clustering coefficient, characteristic path length, eccentricity, global efficiency, diameter, and radius. The analyses were adjusted for covariates: age, years of education, and sex. To ensure assumptions were met, normality of residuals was tested using Kolmogorov–Smirnov tests. We adopted Belsley collinearity diagnostics (MATLAB R2015a) to assess multicollinearity effects between the estimated network metrics.

## Results

### Inclusion of metrics into integrated networks using correlation and collinearity tests

**FIG. 3.**
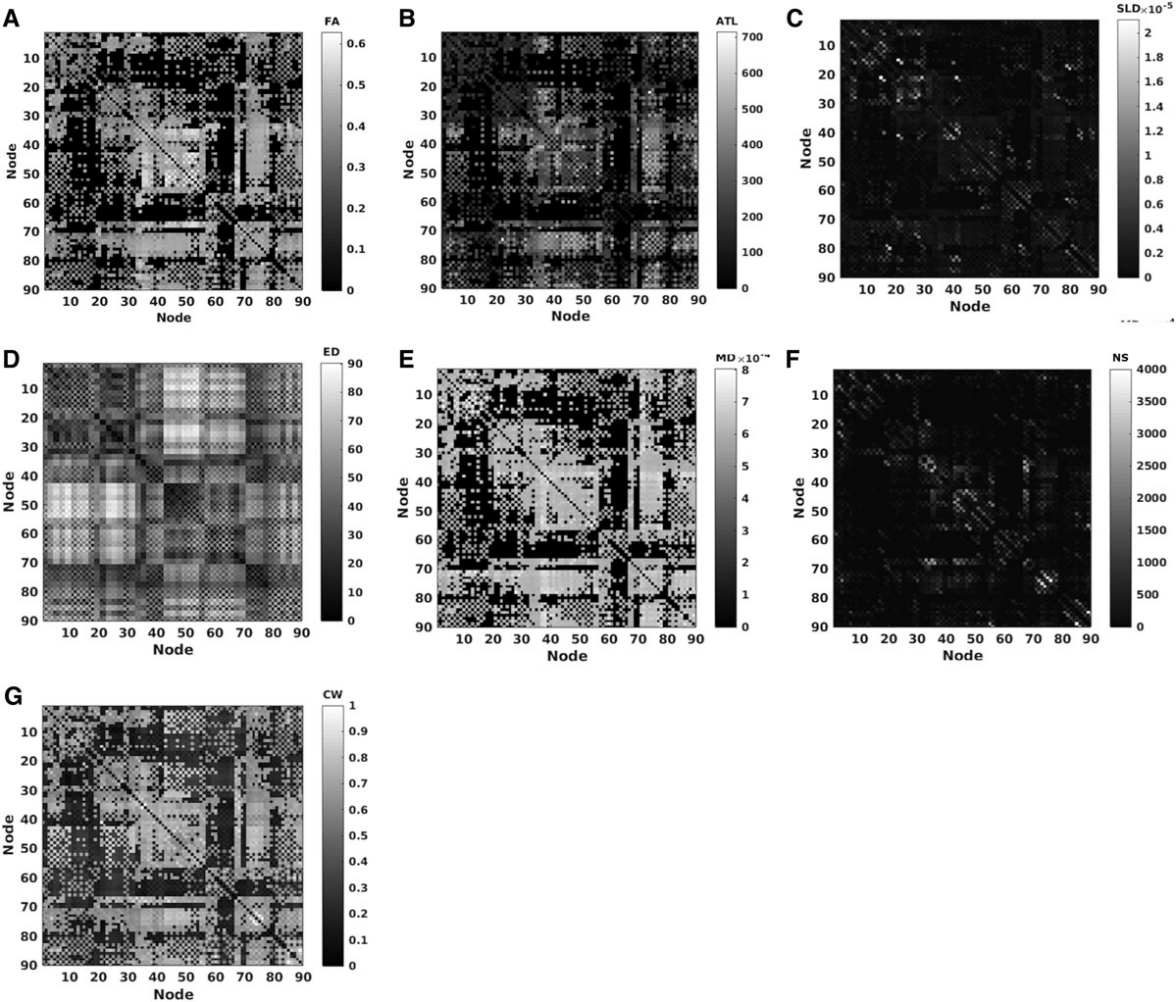
An example of the six individual network measures that were combined into an integrated weighted structural brain network for one participant. **(A)** FA, **(B)** ATL, **(C)** SLD, **(D)** ED, **(E)** MD, and **(F)** NS were combined into an **(G)** integrated weighted structural brain network. ATL, average tract length; CW, connectivity weight; ED, Euclidean distance; MD, mean diffusivity; NS, number of streamlines; SLD, streamline density.

A multicollinearity test was performed on the 10 variables with a default cutoff of 30 for the condition index and 0.5 for proportion of variance decomposition. This analysis revealed multicollinearity between AxD, MD, and RD (Table 3). Correlation coefficients (Table 4) were calculated between all 10 connectivity metrics. We used a cutoff of *R* > 0.6 to flag strong correlations to investigate further. PS, NS, and TV were highly intercorrelated, and for that reason we only included NS in our analysis. AxD, MD, and RD exhibit multicollinearity and both AxD and RD correlated strongly with FA (*R* = 0.6036, *p* < 10^−8^ and *R* = −0.6721, *p* < 10^−8^, respectively)—thus these two metrics were excluded. This resulted in a final inclusion of ATL, SLD, FA, ED, MD, and NS. We reran the correlation and multicollinearity analysis on these metrics and confirmed no strong correlations (Table 4) or multicollinearity. These six metrics were then combined into a single graph (Fig. 3) with an algorithm introduced in our previous study (Dimitriadis et al., 2017b).

**Table 3.** Belsley Collinearity Diagnostics Results for Diffusion-Weighted Magnetic Resonance Imaging Connectivity Matrices

sValue	CondIdx	AxD	ATL	ED	FA	MD	NS	PS	RD	SLD	TV
2.7153	1	0	0.001	0.0013	0.0001	0	0.0004	0.0005	0	0.0029	0.001
1.3103	2.0722	0	0.0021	0.0047	0.0001	0	0.0074	0.0104	0	0	0.0082
0.7989	3.399	0	0.0073	0.0065	0	0	0.0002	0.0005	0	0.4447	0.0034
0.3047	8.91	0	0.0299	0.3087	0.0001	0	0.0155	0.1257	0	0.0001	0.3775
0.2837	9.5698	0	0.0521	0.3839	0.0063	0	0.0018	0.043	0	0.3094	0.1527
0.2258	12.0263	0	0.7593	0.1808	0	0	0.0041	0.1228	0	0.1427	0.1425
0.1587	17.1051	0	0.0497	0.0493	0.0007	0	0.969	0.693	0	0.0078	0.3067
0.1433	18.9544	0	0.085	0.0034	0.2084	0	0.0016	0.0037	0	0.0732	0.0074
0.0414	**65.5353**	0	0.0135	0.0614	**0.7839**	0	0	0.0003	0	0.0192	0.0005
0	**2.26E+14**	1	0	0.0001	0.0004	**1**	0	0	**1**	0	0

Belsley collinearity diagnostics run across the dMRI metrics demonstrating multicollinearity between AxD, MD, and RD. The bold numbers identify metrics that meet our exclusion criteria, condition index >30, and variance decomposition >0.5.

Abbreviations of the dMRI metrics are defined in Table 2.

CondIdx, condition index; sValue, singular values.

**Table 4.** Correlation Coefficients (*R*) Determined by MATLAB (corrcoef) Between the Individual Connectivity Metrics (Abbreviations Defined in Table 2)

	ATL	AxD	SLD	FA	ED	MD	NS	PS	RD	TV
ATL	1									
AxD	0.4033	1								
SLD	−0.5558	−0.2418	1							
FA	0.4772	**0.6036**	−0.4588	1						
ED	0.5727	0.1300	−0.4509	0.2474	1					
MD	0.1638	**0.7722**	0.0311	−0.0300	−0.0089	1				
NS	−0.2631	−0.0373	0.1426	0.0101	−0.4070	−0.0680	1			
PS	−0.2599	−0.0270	0.1253	0.0262	−0.4122	−0.0679	**0.9564**	1		
RD	−0.1635	0.1630	0.2989	**−0.6721**	−0.1485	**0.7527**	−0.0669	−0.0775	1	
TV	−0.0986	0.0446	0.0322	0.0871	−0.3529	−0.0146	**0.9021**	**0.8600**	−0.0689	1

	ATL	SLD	FA	ED	MD	NS				
After excluding AxD, PS, RD, and TV										
ATL	1									
SLD	−0.5558	1								
FA	0.4772	−0.4588	1							
ED	0.5727	−0.4509	0.2474	1						
MD	0.1638	0.0311	−0.0300	−0.0089	1					
NS	−0.2631	0.1426	0.0101	−0.4070	−0.0680	1				

Bold numbers identify intercorrelations with an *R* > 0.6. The lower half of the table shows reduced intercorrelation coefficients after the analysis has been rerun with AxD, PS, RD, and TV excluded.

### Exclusion of mean eccentricity from further analyses

Belsley collinearity diagnostics applied over the adopted set of network metrics flagged multicollinearity between diameter and mean eccentricity using whole-brain network measures (Table 5). We therefore excluded the eccentricity from further analyses and kept the diameter, which in combination with the radius informs us about the lower (radius) and upper limits (diameter) of eccentricity.

**Table 5.** Belsley Collinearity Diagnostics Results: Network Measures

sValue	CondIdx	Diameter	Efficiency	Lambda	Radius	Clustering coefficient	Eccentricity
2.4172	1.0000	0.0001	0.0003	0.0001	0.0000	0.0004	0.0000
0.3748	6.4489	0.0006	0.0257	0.0038	0.0010	0.0374	0.0003
0.0890	27.1550	0.0440	0.3625	0.1359	0.0011	0.5189	0.0002
0.0812	29.7691	0.0640	0.5520	0.1607	0.0000	0.3796	0.0018
0.0395	**61.2534**	0.2325	0.0107	0.2996	**0.7250**	0.0146	0.0004
0.0205	**118.0614**	**0.6589**	0.0487	0.4000	0.2729	0.0492	**0.9972**

This table demonstrates multicollinearity between whole-brain diameter and mean eccentricity when assessed with Belsley collinearity diagnostics. Bold numbers indicate metrics that meet our exclusion criteria, condition index >30, and variance decomposition >0.5.

Lambda, characteristic path length.

### Whole-brain analysis

Kolmogorov–Smirnov tests for normality revealed non-Gaussian distributions for all network measures (*p* < 0.05, Table 6 and Supplementary Fig. S1). To alleviate this, diameter, characteristic path length, and radius were log transformed to reduce positive skew, and efficiency and clustering coefficients were squared to reduce negative skew. After data cleaning, the diameter, characteristic path length, and radius mimicked normal distributions when assessed by Kolmogorov–Smirnov tests (*p* > 0.05), but efficiency (skew = −0.688, SE_skew_ = 0.192, kurtosis = 0.516, SE_kurtosis_ = 0.381) and clustering coefficients (skew = −0.154, SE_skew_ = 0.192, kurtosis = 1.639, SE_kurtosis_ = 0.381) were non-normal. Despite the latter, the analysis was continued as the graphs, when inspected, appeared improved beyond the original in regard to skew (Supplementary Fig. S1), and therefore, we decided that the data complied with general linear model assumptions despite formally failing the tests. One extreme outlier was present in the diameter data after transformation (defined as >3 × interquartile range), and thus, we excluded this participant from further whole-brain analyses. Omnibus multivariate analyses revealed no significant main or interaction effects (*p* > 0.05, Table 7), suggesting that there were no differences in whole-brain network measures between individuals who carry *APOE4* versus noncarriers, have an FH versus no FH, and obese versus healthy WHR.

**Table 6.** Kolmogorov–Smirnov Test Results for the Whole-Brain, Default Mode Network and Visual System

Standardized residual	Statistic	DF	*p*
Before data cleaning			
Whole			
Diameter	0.078	161	0.019
Global efficiency	0.114	161	0.000
Characteristic path length	0.082	161	0.010
Radius	0.083	161	0.008
Clustering coefficient	0.115	161	0.000
DMN			
Diameter	0.118	161	0.000
Global efficiency	0.200	161	0.000
Characteristic path length	0.158	161	0.000
Radius	0.120	161	0.000
Clustering coefficient	0.146	161	0.000
Visual			
Diameter	0.128	161	0.000
Global efficiency	0.128	161	0.000
Characteristic path length	0.124	161	0.000
Radius	0.135	161	0.000
Clustering coefficient	0.086	161	0.006
After data cleaning			
Whole			
Logged diameter	0.067	160	0.080
Squared global efficiency	0.091	160	0.003
Logged characteristic path length	0.047	160	0.200
Logged radius	0.053	160	0.200
Squared clustering coefficient	0.083	160	0.009
DMN			
Logged diameter	0.059	151	0.200
Squared global efficiency	0.151	151	0.000
Logged characteristic path length	0.084	151	0.010
Logged radius	0.060	151	0.200
Clustering coefficient	0.123	151	0.000
Visual			
Logged diameter	0.091	161	0.003
Squared global efficiency	0.108	161	0.000
Logged characteristic path length	0.082	161	0.009
Logged radius	0.088	161	0.004
Squared clustering coefficient	0.060	161	0.200

Lack of normality of standardized residuals assessed with Kolmogorov–Smirnov tests of the whole-brain, DMN and visual system. The lower part of the table demonstrates how the normality of the metrics is improved after data cleaning (removing outliers and transforming the data). *p* Values are reported to 3 decimal places.

DF, degrees of freedom; DMN, default mode network.

**Table 7.** Multivariate Results

Effect	F	DF	*p*
Whole-brain analysis			
Intercept	48.648	5, 145	0.000
Sex	0.841	5, 145	0.523
Age	1.325	5, 145	0.257
Years of education	1.904	5, 145	0.097
FH	1.307	5, 145	0.264
*APOE4*	0.351	5, 145	0.881
WHR	0.981	5, 145	0.432
FH × *APOE4*	1.019	5, 145	0.409
FH × WHR	0.532	5, 145	0.752
*APOE4* × WHR	0.533	5, 145	0.751
FH × *APOE4* × WHR	1.666	5, 145	0.147
DMN analysis			
Intercept	84.361	5, 136	0.000
Sex	1.315	5, 136	0.261
Age	1.867	5, 136	0.104
Years of education	1.010	5, 136	0.414
FH	1.523	5, 136	0.187
*APOE4*	0.924	5, 136	0.567
WHR	0.201	5, 136	0.961
FH × *APOE4*	0.242	5, 136	0.242
FH × WHR	0.733	5, 136	0.733
*APOE4* × WHR	0.940	5, 136	0.940
FH × *APOE4* × WHR	0.444	5, 136	0.444
Visual system analysis			
Intercept	73.555	5, 146	0.000
Sex	0.534	5, 146	0.750
Age	1.989	5, 146	0.084
Years of education	1.314	5, 146	0.261
FH	0.351	5, 146	0.881
*APOE4*	0.901	5, 146	0.482
WHR	1.179	5, 146	0.322
FH × *APOE4*	0.284	5, 146	0.921
FH × WHR	0.986	5, 146	0.429
*APOE4* × WHR	1.365	5, 146	0.241
FH × *APOE4* × WHR	2.089	5, 146	0.070

There were no significant differences in network measures as a function of risk factors: FH, *APOE4*, and WHR across the whole-brain, DMN or a control subnetwork (visual system). *p* Values are reported to 3 decimal places.

*F*, *F* statistic.

### Subnetwork analyses

We then investigated whether any individual differences were occurring at a subnetwork level.

#### DMN analysis

Kolmogorov–Smirnov tests revealed non-normality for all network measures calculated from the DMN integrated graphs (Table 6 and Supplementary Fig. S2). To correct for this, the diameter, characteristic path length, and radius were log transformed and the efficiency was squared. Despite being non-Gaussian as determined by Kolmogorov–Smirnov tests, the distribution of residuals for mean clustering coefficients was not too heavily skewed. We identified nine extreme outliers (>3 × interquartile range) within efficiency data and one within clustering coefficients, and thus, these were removed from the analysis. After data cleaning, the characteristic path length (skew = 0.508, SE_skew_ = 0.197, kurtosis = 0.516, SE_kurtosis_ = 0.381), efficiency (skew = −1.522, SE_skew_ = 0.197, kurtosis = 3.106, SE_kurtosis_ = 0.392), and clustering coefficients (skew = −0.599, SE_skew_ = 0.197, kurtosis = 1.131, SE_kurtosis_ = 0.392) were not formally normal when reassessed with Kolmogorov–Smirnov tests, however, the analysis was continued (Supplementary Fig. S2) as not to lose value in our raw data, as a result of another round of data cleaning. Multivariate analyses revealed no significant effects (*N* = 151, *p* > 0.05, Table 7), suggesting that there are no differences in DMN measures as a result of risk-factor profile.

#### Visual network analysis

Kolmogorov–Smirnov tests revealed non-normality for all six network measures for the visual system (Table 6 and Supplementary Fig. S3). Following the same process as before, the diameter, characteristic path length, and radius were log transformed and the efficiency and clustering coefficients were squared. No outliers were identified in the transformed metrics. Diameter (skew = 0.852, SE_skew_ = 0.192, kurtosis = 1.004, SE_kurtosis_ = 0.38), characteristic path length (skew = 0.0614, SE_skew_ = 0.191, kurtosis = 1.385, SE_kurtosis_ = 0.38), radius (skew = 0.751, SE_skew_ = 0.191, kurtosis = 1.164, SE_kurtosis_ = 0.38), and efficiency (skew = −0.956, SE_skew_ = 0.191, kurtosis = 1.241, SE_kurtosis_ = 0.38) were non-normal, however, the analysis was continued (Supplementary Fig. S3), with a sample size of 161, as the distributions were improved beyond the untransformed metrics to a point that we believe meets the underlying assumptions of the analysis. The general linear model (*N* = 161) revealed no significant multivariate effects (Table 7).

### Analysis of network hubs in the whole brain

**FIG. 4.**
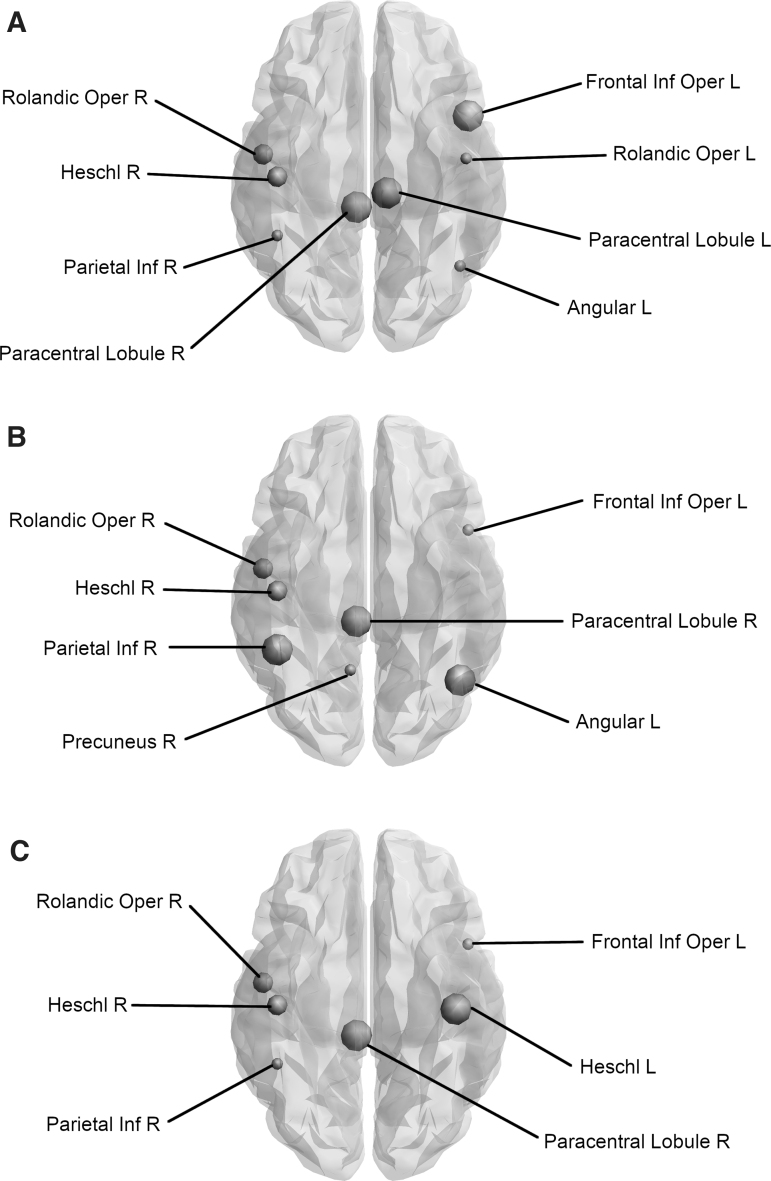
Nodes identified as hubs, change dependent on risk factor profile. This figure shows the changes in nodes defined as hub regions, when you transition from a low-risk group to a high-risk group. A size scale is used to define hub changes, large symbols indicate gained hubs whereas small symbols represent those hubs which are lost. The intermediate size indicates hubs that remain. **(A)** Comparing individuals without an FH with those with a positive FH indicates that two hubs remain unchanged, whereas three are gained and three are lost. **(B)** Comparing *APOE4* noncarriers with carriers results in a gain of three hubs, loss of two hubs, but leaves two hubs unchanged. **(C)** In comparison with healthy individuals, obese participants gained two hubs, lost two hubs, and two hubs remain *APOE4*, apolipoprotein-E ɛ4; FH, family history of dementia.

Replicator dynamics identified hubs consistent across the individual risk factor groups. Individuals with no FH (*N* = 104) had hubs located in the left and right Rolandic operculum, right inferior parietal gyrus, left angular gyrus, and right Heschl's gyrus, whereas individuals with a positive FH (*N* = 59) had hubs at the right Rolandic operculum, left inferior frontal gyrus opercular part, left and right paracentral lobule, and the right Heschl's gyrus (Fig. 4). Individuals who had a healthy WHR (*N* = 63), and thus considered at less risk of developing AD, had hubs within the left inferior frontal gyrus opercular part, right Rolandic operculum, right inferior parietal gyrus, and right Heschl's gyrus, whereas individuals who were centrally obese (*N* = 102) had hubs within the right Rolandic operculum, right paracentral lobule, and both left and right Heschl's gyri (Fig. 4). Participants who were negative for the *APOE4* allele (and thus considered low risk) had hubs in the left inferior frontal gyrus opercular part, right Rolandic operculum, right precuneus, and right Heschl's gyrus (*N* = 100), whereas *APOE4*-positive individuals (*N* = 64) had hubs in the right Rolandic operculum, right inferior parietal gyrus, left angular gyrus, right paracentral lobule, and right Heschl's gyrus (Fig. 4).

To summarize the above-described pattern, the right Rolandic operculum and Heschl's gyrus remain present as hubs regardless of risk factor. In contrast, at-risk individuals (obese, positive FH, and *APOE4* carriers) consistently have a hub in the right paracentral lobule, which is absent in their respective low-risk group (Table 8 and Fig. 4).

**Table 8.** Hub Changes As a Function of Risk Factor

Risk factor change	Hubs that remain	Hubs that are lost	Hubs that are gained
Whole-brain network			
Negative FH → Positive FH	Right Rolandic operculum Right Heschl's gyrus	Left Rolandic operculum Right inferior parietal gyrus Left angular gyrus	Left inferior frontal gyrus opercular part Left paracentral lobule Right paracentral lobule
*APOE4* noncarrier → *APOE4* carrier	Right Rolandic operculum Right Heschl's gyrus	Left inferior frontal gyrus opercular part Right precuneus	Right inferior parietal gyrus Left angular gyrus Right paracentral lobule
WHR healthy → WHR obese	Right Rolandic operculum Right Heschl's gyrus	Left inferior frontal gyrus opercular part Right inferior parietal gyrus	Right paracentral lobule Left Heschl's gyrus
DMN			
Negative FH → Positive FH	Left inferior frontal gyrus opercular part Right inferior frontal gyrus opercular part Right inferior parietal gyrus Left angular gyrus	N/A	Right precuneus
*APOE4* noncarrier → *APOE4* carrier	Left inferior frontal gyrus opercular part Right inferior frontal gyrus opercular part Right inferior parietal gyrus Left angular gyrus	Right precuneus	Left inferior parietal gyrus
WHR healthy → WHR obese	Left inferior frontal gyrus opercular part Right inferior frontal gyrus opercular part Right inferior parietal gyrus Left angular gyrus	N/A	N/A
Visual subnetwork			
Negative FH → Positive FH	Right calcarine fissure Right middle occipital lobe Right inferior occipital lobe	N/A	N/A
*APOE4* noncarrier → *APOE4* carrier	Right calcarine fissure Right middle occipital lobe Right inferior occipital lobe	N/A	N/A
WHR healthy → WHR obese	Right calcarine fissure Right middle occipital lobe Right inferior occipital lobe	N/A	N/A

For the whole-brain analysis (top): The right Rolandic operculum and right Heschl's gyrus remain constant when switching from a low-risk—no FH, no *APOE4* allele, healthy WHR score—to a high-risk group (FH, *APOE4*, centrally obese). Whereas the right paracentral lobule is consistently gained. Furthermore, a few more hubs are gained or lost, although inconsistent across risk factor groups. In the DMN analysis (middle): Individuals with an FH had a hub in the right precuneus in contrast to those with no FH. Conversely, this hub is lost between individuals with no *APOE4* in comparison with those who carry *APOE4* and instead a hub is gained within the left inferior parietal gyrus. In the visual subnetwork analysis (bottom): Hubs within the right calcarine fissure, right middle occipital lobe, and right inferior occipital lobe remained in all risk factor manipulations.

### Analysis of internally important nodes/hubs in the DMN

Hubs were identified within the left and right opercular parts of the inferior frontal gyrus, right inferior parietal gyrus, and left angular gyrus in individuals with no FH, whereas individuals with FH had hubs within the left and right opercular parts of the inferior frontal gyrus, right inferior parietal gyrus, left angular gyrus, and right precuneus. Both individuals of healthy WHR and individuals who were obese had hubs within the left and right opercular parts of the inferior frontal gyrus, right inferior parietal gyrus, and left angular gyrus. In participants without the *APOE4* allele, hubs were identified in the left and right opercular parts of the inferior frontal gyrus, right inferior parietal gyrus, left angular gyrus, and right precuneus, whereas individuals who carry *APOE4* had hubs within the left and right opercular parts of the inferior frontal gyrus, left and right inferior parietal gyri, and left angular gyrus.

As opposed to the analysis of the whole brain, there were no consistent differences of hubs within the DMN as a result of risk factor profile. Individuals without an FH compared with those with an FH gained a hub within the right precuneus, whereas this hub was lost in the transition between *APOE4* noncarriers and carriers and instead they gained a hub in the left inferior parietal gyrus (Table 8).

### Analysis of internally important nodes/hubs in the visual subnetwork

Replicator dynamics identified internally important nodes within the right calcarine fissure, right middle occipital lobe, and right inferior occipital lobe of the visual subnetwork. Each of these hubs was identified regardless of risk factor profile, suggesting that AD risk has no effect on hubs within the visual network (Table 8).

## Discussion

To the best of our knowledge, our study investigated for the first time the effects of *APOE4* genotypes, central obesity, and FH on the graph theoretical metrics of structural brain networks derived via tractography, in cognitively healthy adults. The advantage of our analysis methods over conventional structural network analyses lies in the use of integrated structural network matrices, which combine, in a data-driven manner, multiple metrics of the white matter tracts, rather than arbitrarily using one metric. This means that more information is included in the individual structural network matrices.

Graph theoretical metrics expressing segregation and integration of each participant's structural brain connectome were calculated for the whole brain and for two subnetworks, the DMN (which is known to be impaired in AD) and the visual network (used here as a control network). Multivariate analyses revealed no significant effects for either whole brain or for the subnetworks, which suggests that there were no differences in network measures for any of the risk factors (*APOE4*, FH, or central obesity). This interesting finding, which indicates that the integration and segregation properties of these structural networks were preserved in asymptomatic individuals at heightened risk of developing AD, could point to a possible compensatory mechanism that leads to minimal functional disruption (as indicated by the normal cognitive abilities of our sample). We note, however, that it is not known when, or indeed if, any of these individuals would develop AD. Cortical thickness-based structural brain networks, which reflect different organizational properties to the tractography-derived networks used in our analysis, demonstrated altered properties in subjects with mild cognitive impairment (MCI) and AD compared with healthy controls following the progress of the disease (Zhou and Liu, 2013). In addition, Brown and colleagues (2011) found that tractography-derived structural brain networks in older *APOE4* carriers exhibited loss of local interconnectivity in contrast to those of older noncarriers, and that the carriers had impaired memory abilities as well. Finally, Ma and colleagues (2017) found that structural brain connectivity was disrupted in adults (older than 55 years of age) as a result of an interaction between *APOE4* status and developed MCI, more so than it was for *APOE4* carriers only. These findings may suggest that structural connectivity changes are not present in cognitively healthy individuals at risk, and reflect a manifestation of established disease and/or of older age.

Looking at the hubs of the whole-brain structural networks of low-risk versus high-risk individuals, we identified that the three subgroups of high-risk individuals (centrally obese, positive FH, and positive *APOE4*) when compared with individuals in the respective low-risk groups (normal WHR, negative FH, and negative *APOE4*) consistently exhibited a hub in the right paracentral lobule. Importantly, there were no consistent differences of hubs within the DMN and visual network as a result of risk factor profile. The paracentral lobule is located on the medial surface of the cerebral hemisphere and includes parts of both the frontal and parietal lobes. It has gyral projections to the medial frontal gyrus, cingulate sulcus, and precuneus and sulcal projections to the paracentral, cingulate, precentral sulci, and the pars marginalis of cingulate sulcus. The paracentral lobule controls motor and sensory innervations of the contralateral lower limb. In a recent study, widespread cortical thinning in the left hemisphere regions, including the pericalcarine cortex, supramarginal gyrus, cuneus cortex, lateral occipital cortex, precuneus cortex, fusiform gyrus, superior frontal gyrus, lateral occipital cortex, entorhinal cortex, inferior parietal cortex, isthmus-cingulate cortex, postcentral gyrus, superior parietal cortex, caudal middle frontal gyrus, insula cortex, precentral gyrus, and paracentral lobule, was observed in patients with AD compared with normal controls (Yang et al., 2019). Another structural MRI study on nondemented older subjects revealed a modulation of the cortical thickness covariance between the left parahippocampal gyrus and left medial cortex, supplementary motor area, the left medial superior frontal gyrus, and paracentral lobule driven by the interaction of the rs405509 genotype and age (Chen et al., 2015). In a previous CARDS analysis on the same cohort, we explored the impact of *APOE4*, FH and WHR on white matter microstructure (Mole et al., 2020a). Individuals with the highest genetic risk (FH+ and *APO-E4*) showed a reduced macromolecular proton fraction (MPF) from quantitative magnetization transfer in the right parahippocampal cingulum associated with obesity. In addition, *APOE4*-related MPF reductions were apparent in the left thalamus (Mole et al., 2020b). Furthermore, Rs405509 is an AD-related polymorphism located in the *APOE* promoter region that regulates the transcriptional activity of the *APOE* gene. Abnormal structural brain connectivity was identified between the angular gyrus, superior parietal gyrus, precuneus, posterior cingulum, putamen, precentral gyrus, postcentral gyrus, and paracentral lobule in elders with subjective cognitive decline compared with healthy controls (Kim et al., 2019). These aberrant structural connections were also associated with cognitive scores.

In addition to MRI, PET imaging has identified reduced metabolism in the parietal areas in both *APOE4* carriers with MCI (Paranjpe et al., 2019) and clinical AD (Mosconi et al., 2004). Furthermore, magnetoencephalography (MEG) in young healthy *APOE4* carriers (Koelewijn et al., 2019) has identified hyperconnectivity in the right parietal regions, consistent with the here reported findings. Thus, the novel phenotype we have identified can potentially predict the development of symptoms in a longitudinal study of the same cohort, it could be used as an early biomarker of dementia.

### Assessment of our analysis

Our findings would benefit from replication in a larger sample due to the fragmentation of the initial sample into subgroups with the different risk profiles. It would also be beneficial for structural network analyses to include measures that are believed to play a more important role in the functional performance of the brain, such as myelination of the white matter tracts (Messaritaki et al., 2021) and axonal diameter. We finally note that the thresholding of structural connectivity matrices derived from tractography is still an issue of debate. Buchanan and colleagues (2020), Civier and colleagues (2019), and Drakesmith and colleagues (2015) have shown the possible effects of thresholding when different tractography methods are used. In our analysis, we adopted a modest thresholding of five streamlines, to reduce possible false positives.

## Conclusion

In conclusion, our study did not detect any changes in structural brain networks that would imply alterations in the integration and segregation of structural network properties in cognitively healthy individuals with different risk factors. We identified the right paracentral lobule as a hub brain area in high-risk individuals, but not in low-risk individuals. A longitudinal study of the same cohort with the incorporation of functional neuroimaging data could evaluate this phenotype further.

## Supplementary Material

Supplemental data

Supplemental data

Supplemental data

## Notes

^1^

https://github.com/stdimitr/integrated_structural_brain_networks

^2^

https://github.com/stdimitr/Network_Metrics

^3^

https://github.com/stdimitr/consistent_hubs_cohort

## References

[B1] Badhwar AP, Tam A, Dansereau C, et al. 2017. Resting-state network dysfunction in Alzheimer's disease: a systematic review and meta-analysis. Alzheimers Dement (Amst) 8:73–85. 28560308 10.1016/j.dadm.2017.03.007 PMC5436069

[B2] Basser PJ, Pajevic S, Pierpaoli C, et al. 2000. In vivo fiber tractography using DT-MRI data. Magn Reson Med 44:625–632. 11025519 10.1002/1522-2594(200010)44:4<625::aid-mrm17>3.0.co;2-o

[B3] Brown JA, Terashima KH, Burggren AC, et al. 2011. Brain network local interconnectivity loss in aging APOE-4 allele carriers. Proc Natl Acad Sci U S A 108:20760–20765. 22106308 10.1073/pnas.1109038108 PMC3251140

[B4] Buchanan CR, Bastin ME, Ritchie SJ, et al. 2020. The effect of network thresholding and weighting on structural brain networks in the UK Biobank. Neuroimage 211:116443. 31927129 10.1016/j.neuroimage.2019.116443 PMC7085460

[B5] Buckner RL, Sepulcre J, Talukdar T, et al. 2009. Cortical hubs revealed by intrinsic functional connectivity: mapping, assessment of stability, and relation to Alzheimer's disease. J Neurosci 29:1860–1873. 19211893 10.1523/JNEUROSCI.5062-08.2009 PMC2750039

[B6] Bullmore E, Sporns O. 2009. Complex brain networks: graph theoretical analysis of structural and functional systems. Nat Rev Neurosci 10:186–198. 19190637 10.1038/nrn2575

[B7] Bullmore ET, Bassett DS. 2011. Brain graphs: graphical models of the human brain connectome. Annu Rev Clin Psychol 7:113–140. 21128784 10.1146/annurev-clinpsy-040510-143934

[B8] Chen Y, Li P, Gu B, et al. 2015. The effects of an APOE promoter polymorphism on human cortical morphology during nondemented aging. J Neurosci 35:1423–1431. 25632120 10.1523/JNEUROSCI.1946-14.2015 PMC6795261

[B9] Civier O, Smith RE, Yeh CH, et al. 2019. Is removal of weak connections necessary for graph-theoretical analysis of dense weighted structural connectomes from diffusion MRI? Neuroimage 194:68–81. 30844506 10.1016/j.neuroimage.2019.02.039

[B10] Coad BM, Craig E, Louch R, et al. 2020. Precommissural and postcommissural fornix microstructure in healthy aging and cognition. Brain Neurosci Adv 4:239821281989931. 10.1177/2398212819899316 PMC7085915 32219177

[B11] Dai Z, Lin Q, Li T, et al. 2019. Disrupted structural and functional brain networks in Alzheimer's disease. Neurobiol Aging 75:71–82. 30553155 10.1016/j.neurobiolaging.2018.11.005

[B12] Dell'Acqua F, Scifo P, Rizzo G, et al. 2010. A modified damped Richardson-Lucy algorithm to reduce isotropic background effects in spherical deconvolution. Neuroimage 49:1446–1458. 19781650 10.1016/j.neuroimage.2009.09.033

[B13] Dimitriadis SI, Antonakakis M, Simos P, et al. 2017a. Data-driven topological filtering based on orthogonal minimal spanning trees: application to multigroup magnetoencephalography resting-state connectivity. Brain Connect 7:661–670. 28891322 10.1089/brain.2017.0512 PMC6435350

[B14] Dimitriadis SI, Drakesmith M, Bells S, et al. 2017b. Improving the reliability of network metrics in structural brain networks by integrating different network weighting strategies into a single graph. Front Neurosci 11:694. 29311775 10.3389/fnins.2017.00694 PMC5742099

[B15] Dimitriadis SI, Laskaris NA, Tsirka V, et al. 2010. Tracking brain dynamics via time-dependent network analysis. J Neurosci Methods 193:145–155. 20817039 10.1016/j.jneumeth.2010.08.027

[B16] Dimitriadis SI, Messaritaki E, Jones DK. 2020. The impact of graph construction scheme and community detection algorithm on the reliability of community and hub identification in structural brain networks. BioRXiv. DOI: 10.1101/2020.05.07.082271. PMC8356981 34170066

[B17] Drakesmith M, Caeyenberghs K, Dutt A, et al. 2015. Overcoming the effects of false positives and threshold bias in graph theoretical analyses of neuroimaging data. Neuroimage 118:313–333. 25982515 10.1016/j.neuroimage.2015.05.011 PMC4558463

[B18] Folstein MF, Folstein SE, McHugh PR. 1975. “Mini-Mental state.” A practical methos for grading the cognitive state of patients for the clinician. J Psychiatr Res 12:189–198. 10.1016/0022-3956(75)90026-6 1202204

[B19] Hebert LE, Weuve J, Scherr PA, et al. 2013. Alzheimer disease in the United States (2010–2050) estimated using the 2010 census. Am Acad Neurol 80:1778–1783. 10.1212/WNL.0b013e31828726f5 PMC3719424 23390181

[B20] IBM Corp. 2019. IBM SPSS Statistics for Macintosh, Version 26.0. Released 2019. Armonk, NY: IBM Corp.

[B21] Irfanoglu MO, Walker L, Sarlls J, et al. 2012. Effects of image distortions originating from susceptibility variations and concomitant fields on diffusion MRI tractography results. Neuroimage 61:275–288. 22401760 10.1016/j.neuroimage.2012.02.054 PMC3653420

[B22] John M, Ikuta T, Ferbinteanu J. 2017. Graph analysis of structural brain networks in Alzheimer's disease: beyond small world properties. Brain Struct Funct 222:923–942. 27357309 10.1007/s00429-016-1255-4

[B23] Jones DK, Horsfield MA, Simmons A. 1999. Optimal strategies for measuring diffusion in anisotropic systems by magnetic resonance imaging. Magn Reson Med 42:515–525. 10467296

[B24] Kim D, Lee S, Choi M, et al. 2019. Diffusion tensor imaging reveals abnormal brain networks in elderly subjects with subjective cognitive deficits. Neurol Sci 40:2333–2342. 31243597 10.1007/s10072-019-03981-6

[B25] Koelewijn L, Lancaster TM, Linden D, et al. 2019. Oscillatory hyperactivity and hyperconnectivity in young APOE-ɛ4 carriers and hypoconnectivity in Alzheimer's disease. Elife 8:e360311. 10.7554/eLife.36011 PMC6491037 31038453

[B26] Kroenke K, Spitzer RL, Williams JBW. 2001. The PHQ-9: validity of a brief depression severity measure. J Gen Intern Med 16:606–613. 11556941 10.1046/j.1525-1497.2001.016009606.x PMC1495268

[B27] Leemans A, Jeurissen B, Sijbers J, et al. 2009. ExploreDTI: a graphical toolbox for processing, analyzing, and visualizing diffusion MR data. In: *17th Annual Meeting of the International Society for Magnetic Resonance in Medicine*. Hawaii, USA; p. 3537.

[B28] Livingston G, Huntley J, Sommerlad A, et al. 2020. Dementia prevention, intervention, and care: 2020 report of the Lancet Commission. Lancet 396:413–446. 32738937 10.1016/S0140-6736(20)30367-6 PMC7392084

[B29] Lo CY, Wang PN, Chou KH, et al. 2010. Diffusion tensor tractography reveals abnormal topological organization in structural cortical networks in Alzheimer's disease. J Neurosci 30:16876–16885. 21159959 10.1523/JNEUROSCI.4136-10.2010 PMC6634928

[B30] Ma C, Wang J, Zhang J, Chen K, et al. 2017. Disrupted brain structural connectivity: pathological interactions between genetic APOE ɛ4 status and developed MCI condition. Mol Neurobiol 54:6999–7007. 27785756 10.1007/s12035-016-0224-5

[B31] Messaritaki E, Dimitriadis SI, Jones DK. 2019a. Assessment of the reproducibility of structural brain networks derived using different edge-weighting strategies. In: *Proceedings of the 27th Annual Meeting of the ISMRM*. Montreal, QC, Canada; p. 3364.

[B32] Messaritaki E, Dimitriadis SI, Jones DK. 2019b. Optimization of graph construction can significantly increase the power of structural brain network studies. Neuroimage 199:495–511. 31176831 10.1016/j.neuroimage.2019.05.052 PMC6693529

[B33] Messaritaki E, Foley S, Schiavi S, et al. 2021. Predicting MEG resting-state functional connectivity using microstructural information. Netw Neurosci. [Epub ahead of print]; DOI: 10.1162/netn_a_00187. PMC8233113 34189374

[B34] Metzler-Baddeley C, Mole JP, Leonaviciute E, et al. 2019a. Sex-specific effects of central adiposity and inflammatory markers on limbic microstructure. Neuroimage 189:793–803. 30735826 10.1016/j.neuroimage.2019.02.007 PMC6435101

[B35] Metzler-Baddeley C, Mole JP, Sims R, et al. 2019b. Fornix white matter glia damage causes hippocampal gray matter damage during age-dependent limbic decline. Sci Rep 9:1060. 30705365 10.1038/s41598-018-37658-5 PMC6355929

[B36] Mole JP, Fasano F, Evans J, et al. 2020a. Genetic risk of dementia modifies obesity effects on white matter myelin in cognitively healthy adults. Neurobiol Aging 94:298–310. 32736120 10.1016/j.neurobiolaging.2020.06.014

[B37] Mole JP, Fasano F, Evans J, et al. 2020b. APOE-ɛ4-related differences in left thalamic microstructure in cognitively healthy adults. Sci Rep 10:19787. 33188215 10.1038/s41598-020-75992-9 PMC7666117

[B38] Mosconi L, Nacmias B, Sorbi S, et al. 2004. Brain metabolic decreases related to the close of the ApoE e4 allele in Alzheimer's disease. J Neurol Neurosurg Psychiatry 75:370–376. 14966149 10.1136/jnnp.2003.014993 PMC1738980

[B39] Mukherjee P, Berman JI, Chung SW, et al. 2008a. Diffusion tensor MR imaging and fiber tractography: theoretic underpinnings. Am J Neuroradiol 29:632–641. 18339720 10.3174/ajnr.A1051 PMC7978191

[B40] Mukherjee P, Chung SW, Berman JI, et al. 2008b. Diffusion tensor MR imaging and fiber tractography: technical considerations. Am J Neuroradiol 29:843–852. 18339719 10.3174/ajnr.A1052 PMC8128579

[B41] Nelson HE. 1991. The National Adult Reading Test-Revised (NART-R): Test Manual. Windsor, United Kingdom: National Foundation for Educational Research-Nelson.

[B42] Neumann J, Lohmann G, Derrfuss J, et al. 2005. Meta-analysis of functional imaging data using replicator dynamics. Hum Brain Mapp 25:165–173. 15846812 10.1002/hbm.20133 PMC6871715

[B43] Paranjpe MD, Chen X, Liu M, et al. 2019. The effect of ApoE ɛ4 on longitudinal brain region-specific glucose metabolism in patients with mild cognitive impairment: a FDG-PET study. Neuroimage Clin 22:101795. 30991617 10.1016/j.nicl.2019.101795 PMC6449776

[B44] Parker GD. 2014. Robust Processing of Diffusion Weighted Image Data. Unpublished PhD Thesis. Cardiff University.

[B45] Power JD, Cohen AL, Nelson SM, et al. 2011. Functional network organization of the human brain. Neuron 72:665–678. 22099467 10.1016/j.neuron.2011.09.006 PMC3222858

[B46] Rubinov M, Sporns O. 2010. Complex network measures of brain connectivity: uses and interpretations. Neuroimage 52:1059–1069. 19819337 10.1016/j.neuroimage.2009.10.003

[B47] Tuch DS, Reese TG, Wiegell MR, et al. 2002. High angular resolution diffusion imaging reveals intravoxel white matter fiber heterogeneity. Magn Reson Med 48:577–582. 12353272 10.1002/mrm.10268

[B48] Tzourio-Mazoyer N, Landeau B, Papathanassiou D, et al. 2002. Automated anatomical labeling of activations in SPM using a macroscopic anatomical parcellation of the MNI MRI single-subject brain. Neuroimage 15:273–289. 11771995 10.1006/nimg.2001.0978

[B49] van den Heuvel MP, Sporns O. 2013. Network hubs in the human brain. Trends Cogn Sci 17:683–696. 24231140 10.1016/j.tics.2013.09.012

[B50] Wang L, Roe CM, Snyder AZ, et al. 2012. Alzheimer's disease family history impacts resting state functional connectivity. Ann Neurol 72:571–577. 23109152 10.1002/ana.23643 PMC3490438

[B51] Yang H, Xu H, Li Q, et al. 2019. Study of brain morphology change in Alzheimer's disease and amnestic mild cognitive impairment compared with normal controls. Gen Psychiatr 32:e100005. 31179429 10.1136/gpsych-2018-100005 PMC6551438

[B52] Zhou Y, Liu YW. 2013. Small-world properties in mild cognitive impairment and early Alzheimer's disease: a cortical thickness MRI study Yongxia. ISRN Geriatr 2013:542080. 25414852 10.1155/2013/542080 PMC4235771

