# Supplementary material for: Dementia Risk Factors Modify Hubs but Leave Other Connectivity Measures Unchanged in Asymptomatic Individuals: A Graph Theoretical Analysis
Source: Brain Connect. 2022 Feb 11;12(1):26–40. doi: 10.1089/brain.2020.0935 (PMC8867081; doi:10.1089/brain.2020.0935)
Supplement: Supplemental data [file Supp_FigS1.docx]

**Supplementary Figures**

**S1: Residual distributions before and after data cleaning: whole-brain network**


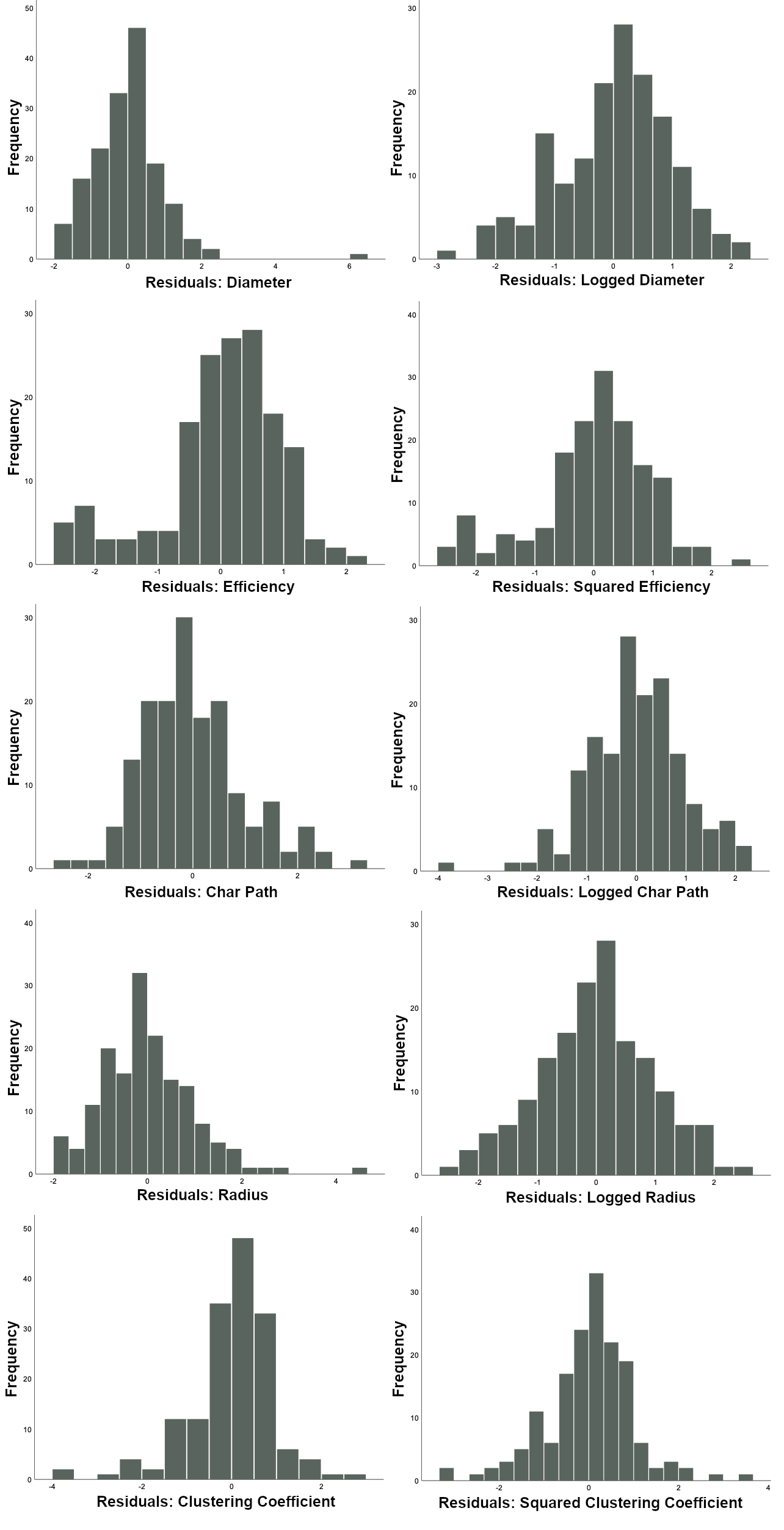


**A)**

**B)**

**C)**

**D)**

**E)**

**F)**

**G)**

**H)**

**I)**

**J)**

**Figure S1. Histograms of standardized residuals for the whole-brain analysis.** **A**, **C**, **E**, **G** and **I** represent the data prior to outlier removal and transforms whereas **B**, **D**, **F**, **H** and **J** show the distributions of the cleaned metrics which were subsequently inputted into the final general linear model. **A)** Diameter was logged to reduce skew (**B**). Efficiency (**C**) was squared (**D**). **E)** Characteristic path length (Char path) was logged (**F**). In addition, **G)** radius was log transformed (**H**). Whereas, clustering coefficients (**I**) were squared (**J**).
