# Supplementary material for: Dementia Risk Factors Modify Hubs but Leave Other Connectivity Measures Unchanged in Asymptomatic Individuals: A Graph Theoretical Analysis
Source: Brain Connect. 2022 Feb 11;12(1):26–40. doi: 10.1089/brain.2020.0935 (PMC8867081; doi:10.1089/brain.2020.0935)
Supplement: Supplemental data [file Supp_FigS3.docx]

**S3: Residual distributions before and after data cleaning: visual sub-network**


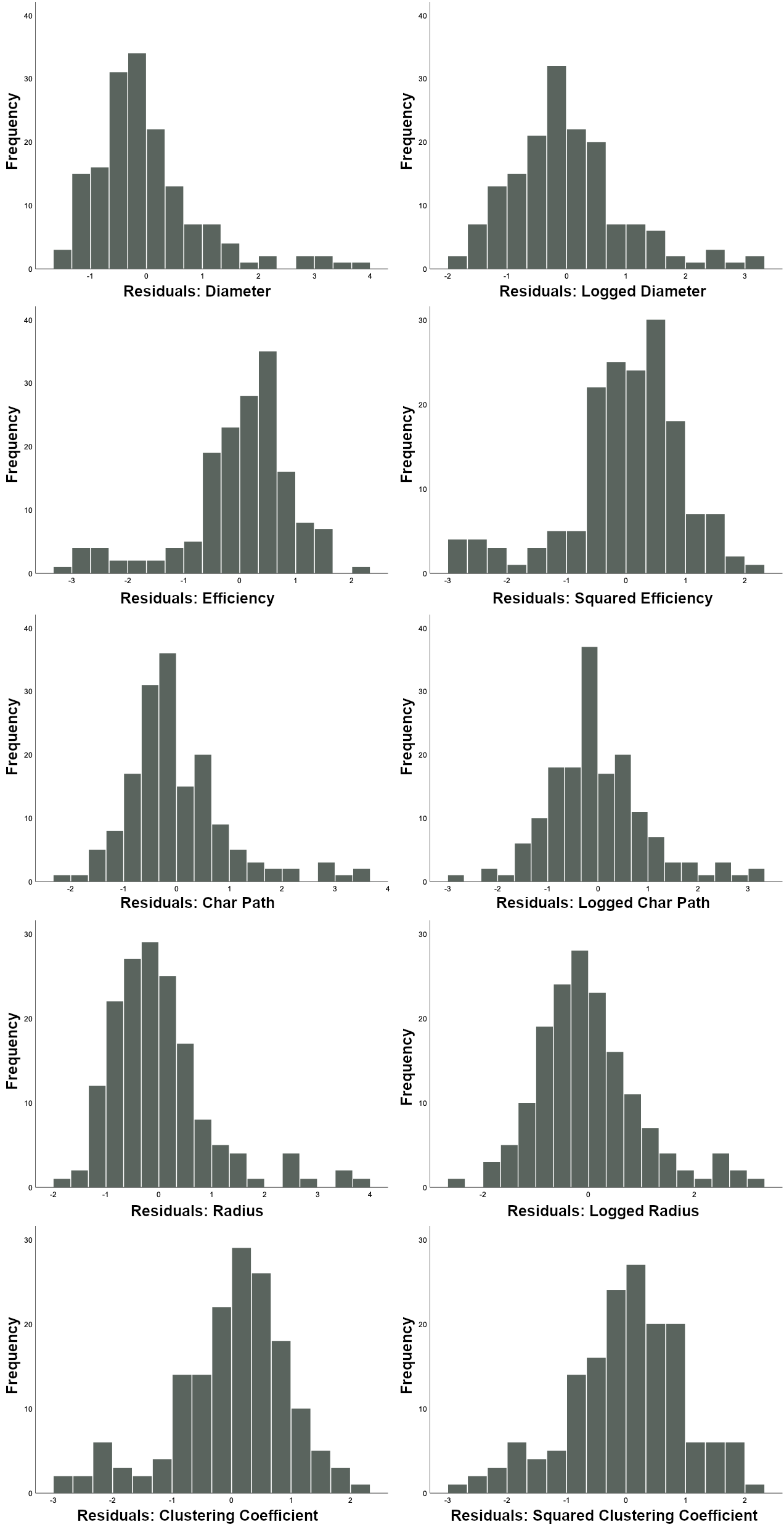


**A)**

**B)**

**C)**

**D)**

**E)**

**F)**

**G)**

**H)**

**I)**

**J)**

**Figure S3. Distributions of standardized residuals for the visual sub-network analysis.** **A**, **C**, **E**, **G** and **I** illustrate data before outlier removal and transforming to reduce skew whereas **B**, **D**, **F**, **H** and **J** represent the residuals of the “cleaned” data. **A)** Diameter was logged to reduce skew (**B**). Efficiency (**C**) was squared (**D**). **E)** Characteristic path length (Char Path) was logged (**F**). In addition, **G)** radius was log transformed (**H**). Whereas, clustering coefficients (**I**) were squared (**J**) to remove skew.
